# Model averaging, optimal inference, and habit formation

**DOI:** 10.3389/fnhum.2014.00457

**Published:** 2014-06-26

**Authors:** Thomas H. B. FitzGerald, Raymond J. Dolan, Karl J. Friston

**Affiliations:** Wellcome Trust Centre for Neuroimaging, UCL Institute of Neurology, University College LondonLondon, UK

**Keywords:** predictive coding, Bayesian inference, habit, interference effect, active inference

## Abstract

Postulating that the brain performs approximate Bayesian inference generates principled and empirically testable models of neuronal function—the subject of much current interest in neuroscience and related disciplines. Current formulations address inference and learning under some assumed and particular model. In reality, organisms are often faced with an additional challenge—that of determining which model or models of their environment are the best for guiding behavior. Bayesian model averaging—which says that an agent should weight the predictions of different models according to their evidence—provides a principled way to solve this problem. Importantly, because model evidence is determined by both the accuracy and complexity of the model, optimal inference requires that these be traded off against one another. This means an agent's behavior should show an equivalent balance. We hypothesize that Bayesian model averaging plays an important role in cognition, given that it is both optimal and realizable within a plausible neuronal architecture. We outline model averaging and how it might be implemented, and then explore a number of implications for brain and behavior. In particular, we propose that model averaging can explain a number of apparently suboptimal phenomena within the framework of approximate (bounded) Bayesian inference, focusing particularly upon the relationship between goal-directed and habitual behavior.

## Introduction

**Figure 1:**
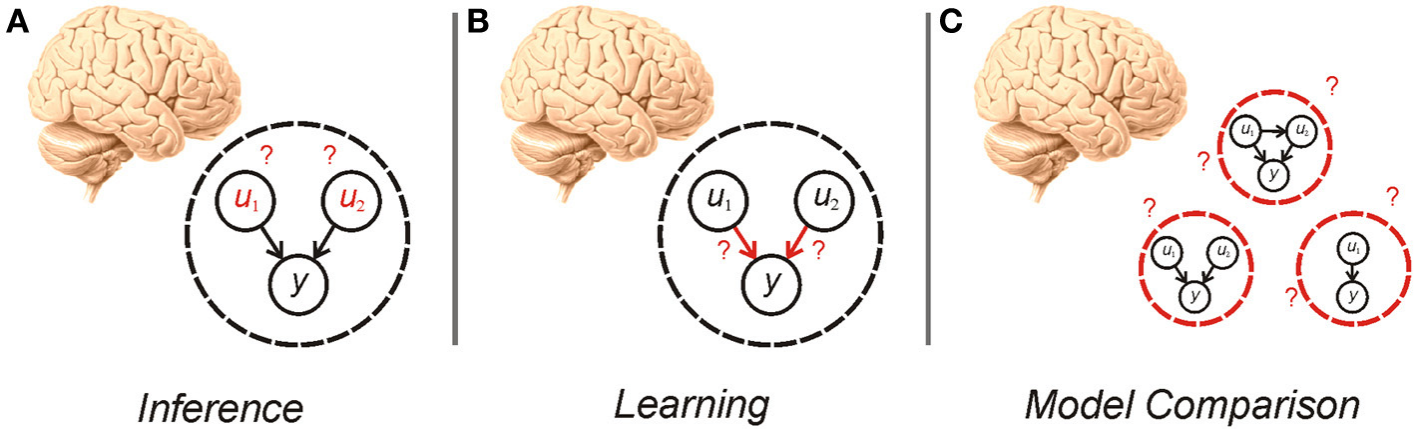
**Cartoon illustrating inference (A), learning (B), and model comparison (C)**. Inference requires an agent to alter its beliefs about the causes (***u*_1_, *u*_2_**) of sensory data (***y***) to maximize model evidence (minimize surprise). Learning also involves the maximization of model evidence, this time through adjustment of the parameters of the model (the mapping between hidden causes and observations). Model comparison involves averaging over—or selecting from—alternative models that can be used for inference and learning.

The idea, first articulated by Helmholtz, that agents perform inference based on a generative model of the world, is the subject of much recent interest in theoretical and experimental neuroscience (Gregory, 1980; Dayan et al., 1995; Rao and Ballard, 1999; Summerfield and Egner, 2009; Friston, 2010; Clark, 2012). In this framework, given a particular model of the world, an agent needs to perform both *inference* about hidden variables and *learning* about the parameters and hyperparameters of the model (Figure 1)—processes that are the focus of much recent study (Friston, 2010; Moran et al., 2013). An equally important consideration however, is determining what model an agent should use in the first place (Hoeting et al., 1999; Penny et al., 2007). This gives rise to an additional tier of uncertainty to those customarily treated in the neuroscientific literature (Yu and Dayan, 2005; Bach and Dolan, 2012)—uncertainty over models. Establishing the best model to use is a pressing concern because, in many situations, the causal structure governing the phenomena of interest is unknown or context dependent (Acuña and Schrater, 2010; Penny et al., 2013). A Bayesian agent needs to consider its own uncertainty about which model is best, and make inferences about evidence for different models, a process known as *model comparison* (Figure 1).

Despite its manifest importance, how the brain adjudicates among models has received little study thus far (though see Courville et al., 2005; Gershman and Niv, 2012; Penny et al., 2013). We first briefly describe Bayesian model comparison (a fuller account is given in the Supplementary Material, Appendix), noting that it depends upon model evidence, which can be approximated using neurobiologically plausible predictive coding schemes (Friston, 2005; Bastos et al., 2012). Crucially, model evidence can be decomposed into an accuracy component—reflecting how well the model predicts observed data—and a (penalizing) complexity component reflecting the computational cost of the model. Thus, Bayes optimal agents seek both to maximize the accuracy of their predictions *and* to minimize the complexity of the models they use to generate those predictions (Jefferys and Berger, 1992). This allows us to formalize heuristic explanations about selection among different models, based on resource costs or their relative reliability (Daw et al., 2005), within a simple and Bayes optimal framework.

The optimal way in which the predictions of different models can be traded off against one another is given by Bayesian model averaging. It is thus highly plausible that this operation is implemented by the brain. We discuss this, together with the relationship between Bayesian model averaging and a related procedure—Bayesian model selection. We then discuss anatomical and behavioral implications of model averaging, and consider several examples of phenomena that can be parsimoniously accounted for by invoking inference over models as a key component of cognitive function. In particular, we focus on the process of habit formation, where, with repeated experience, agents come to rely on simpler models to govern behavior (Dolan and Dayan, 2013). Casting cognition and behavior in this light allows us to reconcile the manifest advantages of performing optimal inference with apparently contradictory phenomena such as bounded rationality (Simon, 1972; Camerer et al., 2004), interference effects (Stroop, 1935; Tucker and Ellis, 2004), and the formation of apparently goal-insensitive habitual behaviors (Yin and Knowlton, 2006).

## Model evidence and model comparison

### Estimating the evidence for a model

We start by outlining the calculations necessary to perform Bayesian model comparison. (these issues are treated more fully in the Supplementary Material, Appendix). First, it is necessary to define a model space containing the set of models {*m_i_:i = 1, …, I*} that are to be compared. Now, given a set of observations *y*, it follows from Bayes theorem that the posterior distribution *p*(*m_i_|y*) over the set of models is given by:

(1) $$p\left(m_{i}\text{​}|\text{​}y\right)\propto p\left(y\text{​}|\text{​}m_{i}\right)p\left(m_{i}\right)$$

This means that model comparison depends on two quantities, the prior probability of the model *p* (*m_i_*), which we will assume here to be equal across models, and the model evidence *p* (*y|m_i_*). This is a key result because the model evidence *p* (*y|m_i_*) is exactly the quantity that is maximized by approximate Bayesian inference and learning. Thus, any agent that performs inference and learning using a particular model of the world necessarily evaluates (implicitly or explicitly) the exact quantity necessary to compare it with other models.

The central importance of model evidence for comparing different models has another important consequence that it is useful to highlight here. Because the model evidence (and approximations to it such as the variational free energy or Bayesian information criterion) contain accuracy and (penalizing) complexity terms (see Supplementary Material, Appendix), the posterior probability of different models also reflects a trade-off between accuracy and complexity. This means that agents will tend to favor simple models, provided they are accurate and, as we shall argue below, this can provide a normative explanation for processes such as habit formation.

Scoring models on more than just the accuracy of their predictions may at first glance seem paradoxical, but in fact the use of a complexity penalty (sometimes called an “Occam factor”) is crucial for optimal inference. This is because it prevents overfitting, a situation where an overly complex model becomes sensitive to noise in the data, limiting its generalization or predictive power for future observations [for a clear discussion of this see (Bishop, 2006) Chapters 1 and 3]. From another perspective, minimizing complexity corresponds to the principle of Occam's razor, where parsimony mandates postulating no more degrees of freedom than are required by the evidence (Jefferys and Berger, 1992).

### Model averaging and model selection

**Figure 2:**
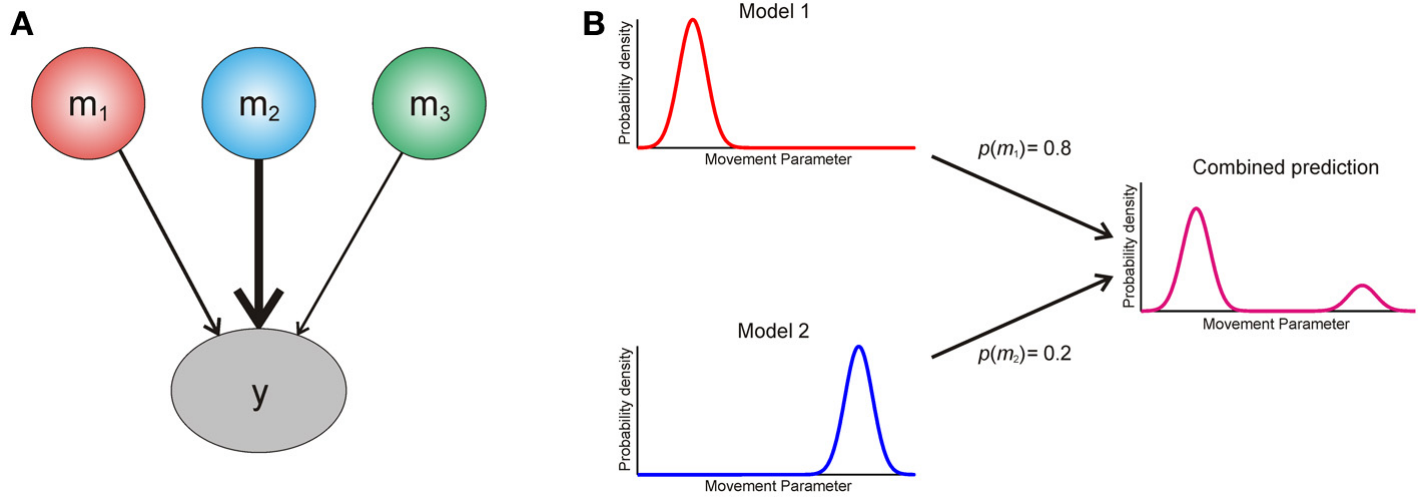
**(A)** Graphical illustration of Bayesian model averaging. To generate a single Bayes optimal prediction about data *y*, the predictions of three models *m*_1−3_ are weighted according to their posterior probabilities [see Equation (A5)]. Here model two has the largest posterior probability, and thus its prediction is weighted most strongly. **(B)** Cartoon explaining interference effects using model comparison. An agent entertains two models of the world, which make different predictions about the probability of making an action based on some movement parameter (x axis). The model probabilities for these are ***p(m_1_)* = 0.8** and ***p(m_2_)* = 0.2** respectively, and the resulting weighted prediction (magenta) shows an interference effect based on this weighted averaging [see Equation (A5)].

We now turn to the question of how an agent should use information from multiple models of its environment. The optimal way in which it can use the predictions of different models is to create a weighted average, with the weight determined by the posterior probability *p* (*m_i_|y*) of each model (Figure 2). This is known as Bayesian model averaging (Hoeting et al., 1999; Attias, 2000; Penny et al., 2007). Intuitively, model averaging is optimal because it uses all available information, weighted according to its reliability, and in this sense it is closely related to optimal integration of information within a single model (Ernst and Banks, 2002). Furthermore, it properly accommodates uncertainty over models in situations where there is no predominant model to call on.

Bayesian model averaging is often contrasted with Bayesian model selection, in which only the best model is used (Stephan et al., 2009). This is suboptimal, but provides a close approximation to model averaging when one model is strongly favored over the rest. In fact, model averaging can always be converted into model selection, as can be seen by changing the softmax parameter implicit in Bayesian model averaging (see Supplementary Material, Appendix). In other words, if one is sufficiently sensitive to differences in model evidence, Bayesian model averaging and selection will yield the same results. This raises the fascinating possibility that, under appropriate conditions, agents can vary the sensitivity of the model comparison they perform (see Model Averaging and Perception). This sensitivity also represents a potential computational phenotype underlying individual differences in normal and pathological behavior.

### Free energy and predictive coding

For certain cases, such as linear Gaussian models, the model evidence can be calculated analytically, but in general its computation is intractable. This necessitates approximate inference, most commonly implemented either using variational methods or sampling [for example Markov Chain Monte Carlo or particle filtering (Bishop, 2006)]. We focus on variational inference here, because it is fast and can (in principle) be implemented within neuronal architectures (Mumford, 1992; Friston, 2005), making it a plausible account of brain function (Friston, 2005; Friston et al., 2013). Here, the model evidence is approximated by the variational free energy, which is minimized during learning and inference (Figure 1). It is easy to see (see Supplementary Material, A4 “Free Energy and Model Averaging”) that model comparison can be performed simply by minimizing the variational free energy across a set of models, suggesting that it could be implemented by the brain.

The most popular and developed account of how the brain might perform variational inference is predictive coding, using hierarchical generative models embodied in the hierarchical structure of the brain (Mumford, 1992; Rao and Ballard, 1999; Friston, 2005, 2008; Bastos et al., 2012) (see Supplementary Material, A5 “Hierarchical Models and Predictive Coding”). Here, model comparison is performed by minimizing the precision-weighted sum of squared prediction errors across a set of models. On this account, if the brain entertains different models of its environment, then these need to make converging top-down predictions of representations in the cortical hierarchy. In some cases, this target might be in primary sensory areas, but it also seems likely that different models may make convergent predictions about higher level representations (the presence or absence of whole objects, for example). A plausible candidate mechanism for weighting the predictions of different models is modulation of the synaptic efficacy of their top-down predictions, either through synchronous gain or through neuromodulators like dopamine. This is an important implementational issue, and one we hope to consider more fully in future work—especially in light of the somewhat surprising finding that at the level of behavior dopamine boosts the influence of complex models at the expense of simpler ones (Wunderlich et al., 2012b).

In summary, we are suggesting that representations at any level of a hierarchical (predictive coding) model are optimized using top-down predictions that represent a Bayesian model average. These predictions are simply the posterior predictions of any given model weighted by posterior beliefs about the model *per se*—beliefs that are directly related to the free energy of each model.

### Related work

A similar approach to the Bayesian model comparison and averaging described here has been employed in the context of supervised learning in mixture of expert models (Jacobs et al., 1991a,b; Jordan and Jacobs, 1994). These consist of a set of expert networks, the outputs of which are weighted by a gating network and combined according to some fixed rule (Jacobs, 1995), which can then be used for classification. Our proposal also bears some resemblance to the MOSAIC model for motor behavior proposed by Kawato and colleagues (Haruno et al., 2001). In MOSAIC, agents are equipped with multiple control modules, which consist of paired forward (predictor) and inverse (controller) models. The weights (“responsibility”) assigned to each module depend upon the accuracy of the forward model predictions in a particular context, and are implemented as prior probabilities according to Bayes rule (Haruno et al., 2001). Motor commands are then the responsibility weighted sum of the outputs of the set of inverse models, and—in situations where more than one control module is assigned a significant responsibility—this may produce similar interference effects to those described above. Compared with both these approaches (at least as they are typically formulated), Bayesian model averaging has the advantage that it considers model evidence, rather than simply model accuracy, and thus meets the demands of optimal inference. In the specific domain of motor control, we note that active (Bayesian) inference formulations require only a single generative model, rather than paired inverse and forward models (Friston, 2011).

Bayesian model averaging itself has been considered in theories of Bayesian conditioning (Courville et al., 2003, 2005); in which models with different numbers of latent causes are entertained by the agent—and their predictions weighted according to the evidence for the different models as in Equation (A5). An interesting and related approach is taken by Gershman and Niv (2012) where instead of averaging the predictions of different models, agents implement a Bayesian non-parametric model (Rasmussen and Ghahramani, 2002; Gershman and Blei, 2012), whose complexity adjusts automatically to the data in hand. These proposals are very close in spirit to the idea presented here, and we note their ability to account for a number of phenomena that are difficult to explain using traditional conditioning models like Rescorla-Wagner learning (Courville et al., 2003, 2005; Gershman and Niv, 2012). It has also recently been proposed that spatial cognition can be explained using approximate Bayesian inference (Penny et al., 2013). In this context, different models correspond to different environments, and thus model comparison can be used as a natural way to perform inference about which environment an agent finds itself in Penny et al. (2013).

## Model averaging and the brain

Here, we briefly consider the implications of Bayesian model averaging for neuroanatomy and development. Much more can (and needs) to be said about this, but our principal focus here is on cognition and behavior, so we will restrict ourselves to some key points:

### Anatomy and development

If agents entertain several models of their environment, in many cases these are likely to co-exist within the same anatomical region. For example, one might imagine that—on encountering a new maze—the hippocampus contains models with many different spatial structures (Blum and Abbott, 1996; Penny et al., 2013), or in other situations that the prefrontal cortex models and compares the evidence for different rules simultaneously (Wallis et al., 2001; Koechlin and Summerfield, 2007). It also seems likely however, given the degree of functional specialization seen in the brain (Zeki et al., 1991)—which itself may arise as a result of approximate Bayesian inference (Friston, 2005; Friston et al., 2013)—that model averaging may call on models encoded in different brain structures (Daw et al., 2005; Graybiel, 2008). One instance of this may underlie the distinction between goal-directed and habitual behavior (Yin and Knowlton, 2006), which we consider in more detail below (for detailed review see Dolan and Dayan, 2013). Another (perhaps related) example might be the apparent competition between hippocampal (largely spatial) and striatal (largely cue-based) mechanisms during instrumental learning (Lee et al., 2008). In general, given that the space of possible models for any situation is potentially uncountable, it makes sense that both evolution and prior experience should act to narrow the space of models entertained, and that particular constraints, such as what features of the environment are considered in the model, should be instantiated in different structures. One can thus think of the brain as performing *selective model averaging* (Heckerman, 1998).

The need to consider different models of the world also provides an interesting perspective on neurodevelopment. Analogous to the way in which model parameters are thought be learnt during development (Fiser et al., 2010; Berkes et al., 2011), one might hypothesize that the posterior distribution over models *p* (*m_i_|y*) becomes increasingly peaked, as learning the best models proceeds. One might further suppose that some form of Occam's window is applied by the brain, in which models below a certain posterior probability are discarded entirely (Madigan and Raftery, 1994). This makes sense in terms of metabolic and other costs and might, in part, explain the decline in cortical volume that occurs with normal ageing (Salat et al., 2004)—since over time agents come to entertain fewer and fewer models. Different degrees of sculpting model space (or else differences in the number or types of models entertained) might then explain regional differences in synaptic regression, such as the observation that neurodevelopmental regression is most pronounced in the prefrontal cortex (Salat et al., 2004). Recently, synaptic regression during sleep has been portrayed in terms of model optimization. In this context, the removal of unnecessary or redundant in synaptic connections (model parameters) minimizes free energy by reducing model complexity (Hobson and Friston, 2012).

### Free energy and resource costs

A widely invoked constraint on the type and complexity of models that animals might build of the world is that imposed by resource or complexity costs. This fits comfortably with minimizing variational free energy—that necessarily entails a minimization of complexity (under accuracy constraints). The link between minimizing thermodynamic free energy and variational free energy has again been discussed in terms of complexity minimization—in the sense that thermodynamic free energy is minimized when complexity is minimized (Sengupta et al., 2013): neuronal activity is highly costly from a metabolic point of view (Laughlin et al., 1998) and for any given phenotype, only a certain volume of neurons (and space) are available within the central nervous system. It is fairly easy to see that—under plausible assumptions about how generative models are implemented neuronally—there will be a high degree of correlation between the complexity of a model and the resource costs of implementing it. Heuristically, having a larger number of models or model parameters would require a larger network of neurons to encode it, which will induce both metabolic and anatomical costs. Another heuristic follows if we assume that the brain uses a predictive coding scheme with explicit biophysical representation of prediction errors. In this context, minimizing the variational free energy will serve to reduce overall neuronal activity (prediction error) and hence metabolic demands. This is because predictive coding minimizes prediction errors throughout the models hierarchy.

While other factors are undoubtedly going to influence the computational cost to an organism of implementing a particular model (there is likely, for example, to be a complex interplay between complexity and different types of cost like time and space costs), there is likely to be a strong relationship between complexity costs (as assessed by the variational free energy) and the metabolic costs to an organism (Sengupta et al., 2013).

## Model averaging and multiple-systems models of decision-masking

A recurring theme in theoretical approaches to human decision-making is that multiple mechanisms are involved in control of behavior, and there is a considerable body of evidence in support of such ideas (Kahneman, 2003; Summerfield et al., 2011; Dolan and Dayan, 2013). We suggest that rather than entirely separate systems competing for control of behavior, the phenomena motivating this tradition can be captured by a view in which anatomically and functionally dissociable networks embody different types of model [which will often have different hierarchical depths—and hence complexity (Kiebel et al., 2008)]. Instead of simple competition behavior can be thought of as resulting from Bayesian model averaging over the predictions of different models. This perspective provides a way to ease the tension between the insight (which goes back at least as far as Plato's tripartite soul) that multiple motivations can be discerned in human behavior, and the manifest advantages of being able to act in a unitary and coherent fashion, particularly if this is approximately Bayes-optimal. We discuss this briefly below, focusing particularly on the interplay between simple and complex models in the control of behavior.

### Habitual and goal-directed behavior

It is well established that animals exhibit both goal-directed behavior, in which action selection is flexible and sensitive to anticipated outcomes, and habitual behavior that is stereotyped and elicited directly by a preceding stimulus or context (Yin and Knowlton, 2006; Graybiel, 2008; Dolan and Dayan, 2013). It has also been shown that the neural substrates of these behaviors are at least partially dissociable (Adams and Dickinson, 1981; Hatfield and Han, 1996; Pickens et al., 2003; Izquierdo et al., 2004; Yin et al., 2004).

Broadly speaking, two mechanisms have been proposed to explain the emergence of habitual behavior. The first posits the existence of separate “model-free” and “model-based” reinforcement learning schemes in different parts of the brain (the dorsolateral striatum and prefrontal cortex) (Daw et al., 2005) that support habitual and goal-directed behavior respectively (Dolan and Dayan, 2013). Which of these two systems controls behavior is determined by their relative uncertainties (Daw et al., 2005), and the emergence of habitual behavior over time results from the model-free system having an asymptotically lower uncertainty than the model-based system. A second hypothesis (though one rarely spelled out explicitly) is that the existence of habits reflects a need to minimize some form of computational, metabolic or attentional cost (Moors and De Houwer, 2006). Once an action has been repeated many times, it comes to be elicited automatically by a particular stimulus or context, removing the need for costly deliberation (these explanations may not be entirely separate from one another, since, as pointed out by one of our reviewers, one reason for the presence of significant noise in the model-based system could be the resource cost of performing complex searches).

Both these hypotheses have much to recommend them, but neither provides a wholly satisfactory account of habit formation. To take the “arbitration by uncertainty” hypothesis first; while the insight that different models of the environment should be traded off against one another—through the accuracy of their predictions—is important, this seems insufficient to explain a transition to habitual behavior in many situations. More specifically, in most (if not all) habit learning experiments, the environment that the agent has to represent is extremely simple (pressing a lever to gain a food pellet, knowing whether to turn left or right in a cross maze). In such contexts it seems *prima facie* implausible that explicit cognitive representations induce a sufficiently large degree of uncertainty so as to be dominated by simple ones [we note that the transition to habitual behavior in Daw et al.'s simulations requires that an arbitrary noise component be used to inflate the uncertainty of the model-based scheme (Daw et al., 2005)]. We suggest that differential uncertainty alone is insufficient to provide a satisfying account of the emergence of habitual behavior. The “cost” hypothesis, by contrast, is inadequate as things stand, because it does not specify in what situations the increased resources necessary for an explicit representation of the environment are justified (or conversely, when the cost of extra complexity is too high).

**Figure 3:**
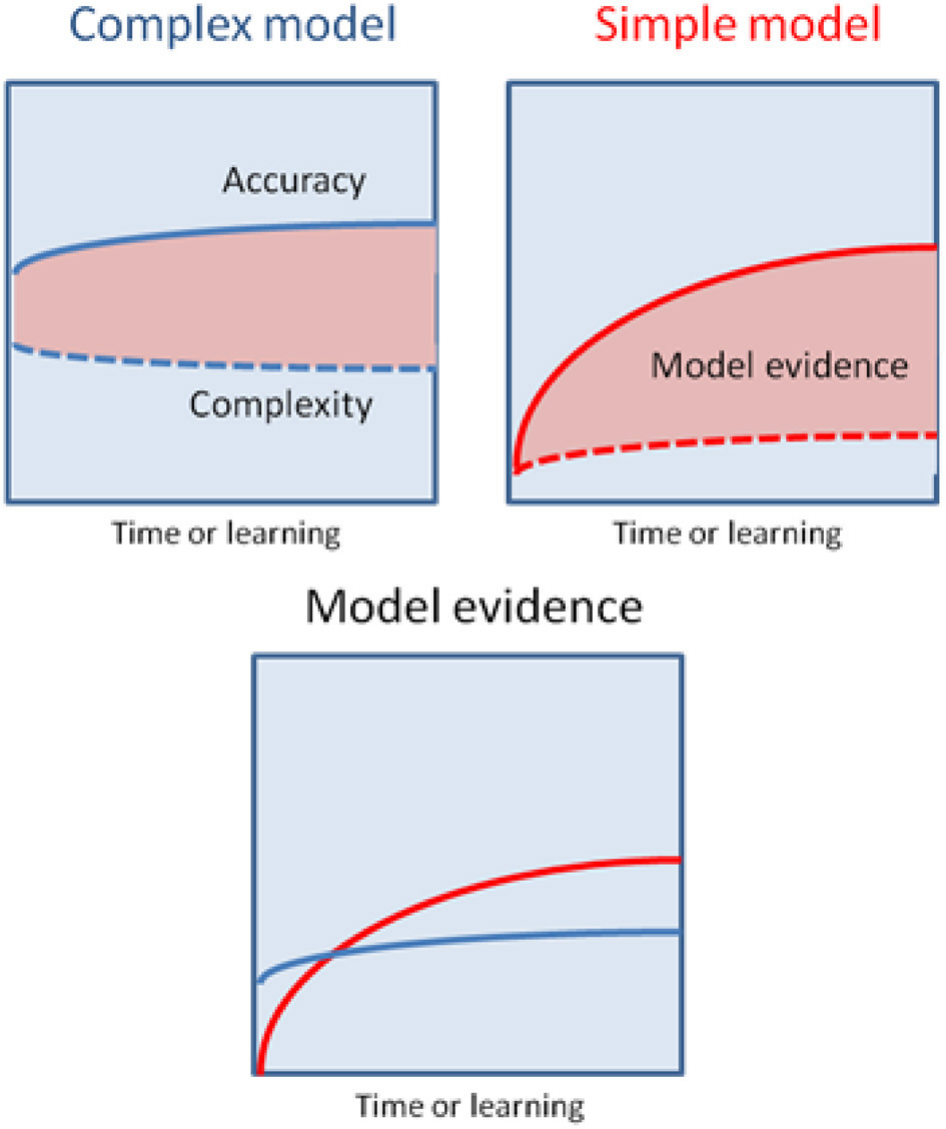
**This schematic illustrates the possibility that a more complex model may have greater model evidence at the start of learning but will then give way to a simpler model as their parameters are optimized**. The upper panels show the learning-related improvement in accuracy and complexity for a complex model (left panel) and a simple model (right panel). The model evidence is shown as the difference (pink areas). The more complex model explains the data more accurately but with a greater complexity cost, that is finessed during the learning. Conversely, the simpler model will always have a lower accuracy but can (with learning) attain greater model evidence—and thereby be selected by Bayesian model averaging as time proceeds and the active inference becomes habitual.

An alternative hypothesis is that habit formation comes about as the result of Bayesian model averaging *between* simple (hierarchically shallow) models and more complicated ones involving richer (hierarchically deep) and more flexible representations of the environment (Kiebel et al., 2008; Wunderlich et al., 2012a). The critical observation is that in Bayesian model comparison models are scored according to both their accuracy and complexity. This means that whilst initially behavior is based largely upon complex models, that are able to generate accurate predictions based on little or no experience, over time simpler models come to predominate, provided their predictions are sufficiently accurate. This will be the case in the stable environments that support habit formation (Figure 3). Bayesian model averaging therefore provides a principled framework that incorporates the insights of both uncertainty- and cost-based explanations, and remedies their defects. On the one hand, model comparison explains why habit formation occurs even in very simple environments that are unlikely to induce significant uncertainty in explicit cognitive representations. The use of simple models will always be favored by the brain, provided those models are accurate *enough*. Informally, this may explain why it is so difficult to suppress learnt habits and other forms of simple stimulus-response behaviors, such as the tendency to approach appetitive stimuli and avoid aversive ones (Guitart-Masip et al., 2011). Very simple models have a very low complexity cost, which means they do not have to be especially accurate in order to be selected for prescribing behavior. On the other hand, invoking model comparison allows us to precisely specify the currency in which different models should be traded off against one another, and provide (in theory at least) a precise account of when increased complexity is justified by increased accuracy, and *vice versa*.

What then, would constitute evidence for the model averaging hypothesis? The strongest grounds, perhaps, are those already described—the extensive body of work characterizing the emergence of habitual behavior, which seems best captured by a view that makes allowance for both model accuracy and model complexity. However, some important recent work using model-based neuroimaging also provides strong support for our hypothesis (Daw et al., 2011; Lee et al., 2014). Both these studies involve asking subjects to perform moderately complex learning tasks, where behavior reflected a combination of both simple (stimulus-response or model-free like) and more complicated (action-outcome or model-based like) models of the environment. Similar findings have been reported by Wunderlich et al. (2012b), Otto et al. (2013) and Smittenaar et al. (2013). In the context of such tasks, model averaging makes two clear predictions. The first is that the control of behavior will be biased toward simple models, once the effects of uncertainty are accounted for. The second is that because the predictions of simple and complex models are unified, there should be evidence of unified (and appropriate weighted) prediction error signals in the brain.

It turns out that both these predictions are borne out by the experimental data. The behavioral modeling presented in Lee et al. strongly suggests that subjects show a bias toward relying on simple models over complex ones (the model-free system over the model-based one in the terminology they employ) (Lee et al., 2014). This is exactly what one would expect if both complexity and accuracy are taken into account. (Daw et al. did not report the results of any similar analysis). Turning to the second prediction Lee et al. report evidence that value signals derived from simple and complex models are integrated in a contextually appropriate way in the ventromedial prefrontal cortex (Lee et al., 2014). Equally importantly, rather than finding separate prediction error signals at outcome presentation for the simple and complex models, Daw et al. instead reported an integrated signal in the ventral striatum, with the strength of expression of the different prediction errors correlated with the relative influence they had over behavior (Daw et al., 2011). Both these findings are precisely in accord with the view that the predictions of simple and complex models are subject to Bayesian model averaging during decision-making. Clearly, the explanation for habit formation on offer is a hypothesis that will need to be tested using simulations and empirical studies; for example, using devaluation paradigms of the sort addressed in Daw et al. (2005)—as suggested by one of our reviewers.

### Habits and behavioral flexibility

The view of habit formation presented here is also consistent with recent discussions that have stressed the flexibility of habitual behavior, and the complex relationship between habitual and goal-directed action (Bernácer and Giménez-Amaya, 2013; Bernácer et al., 2014). Although habitual behavior results from the use of hierarchically shallow models that do not include information about the higher order goals of an organism, they can, under appropriate conditions, instantiate complex links between external stimuli and behavior of the type manifest when performing tasks like driving or playing the piano, rather than just simple stimulus-response mappings. Using shallow models to perform a particular task also frees up neuronal circuits at deeper hierarchical levels, potentially enabling them to be employed in other tasks. Thus, whilst habit formation reduces the flexibility of behavior on a particular task, it may simultaneously increase the overall behavioral repertoire available to the agent. For example, whilst it is difficult for people in the early stages of learning to drive to simultaneously hold a conversation, experienced drivers find this easy. This raises the interesting possibility that, rather than always being antithetical to goal-directed behavior, considered from the perspective of the entire agent, habit formation often enables it. A Bayesian perspective also provides an explanation for how habitual behaviors can be at the same time apparently unconscious and automatic, and yet also rapidly become subject to conscious awareness and goal-directed control when something unexpected occurs (if the brake pedal of the car suddenly stops working, for example) (Bernácer et al., 2014). This occurs because the shallow model generating habitual control of behavior suddenly becomes a poor predictor of current and future sensory information, necessitating the switch to a more complex, flexible model.

### Interference effects, affordances, and pavlovian responses

It has been well documented that human behavior, across a wide variety of domains, shows evidence of what are usually called “interference effects” (Stroop, 1935; Simon et al., 1990; Tipper et al., 1997; Tucker and Ellis, 2004; Guitart-Masip et al., 2011). Typically, these are manifest when subjects are asked to make responses based on one attribute or dimension of a stimulus, but show behavioral impairments, such as slower responding or increased error rates, that can be attributed to a different attribute. Examples of this include the affordance compatibility effect (Tucker and Ellis, 2004), the “Pavlovian” tendency to approach appetitive and avoid aversive stimuli (Dayan, 2008; Guitart-Masip et al., 2011; Huys et al., 2011) and the effect of distractors during reaching (Tipper et al., 1997; Welsh and Elliott, 2004). A closely related phenomenon is that of task switching effects, where subjects' performance is impaired immediately after being asked to swap between performing different tasks (Monsell, 2003).

These effects are generally considered to result from the existence of multiple mechanisms for controlling action (or alternatively, task sets) engaged in more or less blind competition (Dayan, 2008), a scenario virtually guaranteed to produce suboptimal behavior. The arguments presented here suggest another possibility; namely, that such phenomena are the manifestation of agents pursuing a model averaging strategy that is in general optimal, but produces suboptimal behavior in the context of non-ecological experiments (Figure 2). There is a natural parallel with perceptual illusions here, since these result from the application of generally appropriate prior beliefs to situations designed such that these beliefs are inappropriate (Weiss et al., 2002; Shams et al., 2005; Brown and Friston, 2012). To return to the affordance competition and Pavlovian bias effects mentioned above, it seems reasonable to suppose that subjects simultaneously call on a model of their environment induced by the (non-ecological) task demands, and an entrenched (and simpler) model linking stimulus properties like object affordances and stimulus valence to behavioral responding. Since the predictions of these models are averaged, the influence of the simpler models is suppressed, but not entirely attenuated, producing characteristic effects on behavior (Figure 3). This is a hypothesis we will consider more fully in future work. Task switching effects can also naturally be explained, on the hypothesis that models that have recently provided accurate predictions have been accorded a higher posterior probability that is only partially suppressed during switching.

## Model averaging in other cognitive domains

We now turn to considering the consequences of, and evidence for, Bayesian model comparison and averaging in other areas of cognition. We confine our discussion to a small number of examples but we suspect that these ideas may have much broader applicability to other cognitive domains (and perhaps beyond (Friston, 2010, 2012)).

### Model averaging and perception

In certain contexts, perception does indeed show the hallmark of model averaging, namely integration between the predictions of different plausible models. Famous examples of this include the McGurk and ventriloquist effects (McGurk and MacDonald, 1976; Bertelson et al., 2000), in which distinct representations (for example of phonemes in the McGurk effect) are fused into a single percept that is a combination of the two. However, there is also a large literature describing multistability in perception, for example in the face-vase illusion and the Necker cube (Sterzer et al., 2009). Here distinct hypotheses about the world clearly alternate rather than co-existing (Dayan, 1998; Hohwy et al., 2008). A natural explanation for this in the framework we have suggested here is that agents perform apply model averaging with a high sensitivity parameter (see Supplementary Material, A2 “Bayesian Model Averaging”). This effectively implements Bayesian model selection, and ensures that only the predictions of a single preferred model are used. Other explanations are also possible, for example that multistability results from sampling from different models (Gershman et al., 2012) or, as suggested by one of our reviewers, from strong negative covariance between the prior probabilities of different models.

It is unclear precisely why—in some contexts—perception should exhibit integration, and in others multistability, but one attractive possibility is that this is determined by the extent to which an integrated percept is, in itself, plausible. Thus the fused percepts produced by the McGurk and ventriloquist illusions reflect plausible hidden states of the world. By contrast, the intermediate state of a Necker cube, or Rubin's face-vase illusion would be implausible, if not impossible; suggesting that in these contexts agents should preclude perceptual integration by increasing the strictness of their model comparison.

### Experience and bounded rationality

Although in some (particularly perceptual) contexts, human behavior closely approximates the best possible performance (Ernst and Banks, 2002), in many situations it falls well short of this, giving rise to the suggestion that humans are bounded rational decision-makers (Simon, 1972; Kahneman, 2003) rather than perfectly rational; particularly when it comes to economic choice. Bounded rationality means that decision-making is as good as possible, given constraints of one kind or another. A phenomenon is found in theories of social interaction, where it has been shown that humans are able to consider only a (perhaps surprisingly) limited number of levels of recursion on interpersonal choice tasks (Stahl and Wilson, 1995; Camerer et al., 2004; Yoshida et al., 2008; Coricelli and Nagel, 2009).

These specific examples illustrate a more general point. If models are weighted or chosen according to their evidence rather than simply their accuracy, then one should not necessarily expect agents to perform tasks with extremely high levels of accuracy even if they are Bayes optimal. This is because approximate Bayesian inference naturally introduces bounded rationality, since it trades off accuracy (rationality) against complexity (cost). On this view, there are two key determinants of whether agents employ complex models (and hence approximate ideal behavior on tasks where these are necessary). The first is the amount of experience the agent has with a particular task or environment. More experience (equivalent to collecting a large data set in a scientific experiment) allows the increased accuracy of its predictions to outweigh the complexity penalty of a complex model (Courville et al., 2003). The second determinant is the gain in accuracy per observation associated with using the more complex model. This picture fits, at least approximately with what is actually observed in human behavior, where near-ideal performance is often observed in perceptual tasks (which presumably employ models that are used extremely frequently) and suboptimal performance more typically seen in tasks such as abstract reasoning, which are performed less often.

This perspective relates to recent work showing that bounded rationality can be derived from a free energy formulation, where model complexity is introduced by the need to process information in order to perform inference (Ortega and Braun, 2013). Model comparison, as performed by gradient ascent on variational free energy, supplements this insight by explaining how the Bayes-optimal model of the environment arises.

## Other issues

### Where does the model space come from?

One issue we have not touched on is how models are created in the first place. This is a deep and challenging topic, whose proper consideration falls outside the scope of this piece. One easy answer is that the space of possible models is constrained by phylogeny and thus ultimately by natural selection, which can itself be thought of in terms of free energy minimization (Kaila and Annila, 2008). From the perspective of neuroscience, this is at the same time true and unsatisfying. To understand how new models are generated within the lifetime of an organism (and *a fortiori* on the timescale of laboratory experiments), it is interesting to consider structure learning (Heckerman, 1998; Needham et al., 2007; Braun et al., 2010; Tenenbaum et al., 2011). Structure learning deals with the problem of how to infer dependencies between hidden variables, and allows inferences to be drawn about both the specific model structure (Heckerman, 1998; Tenenbaum et al., 2011) and the general structural form (for example a ring, tree or hierarchy) (Kemp and Tenenbaum, 2008) most appropriate for a dataset. From our perspective, this is simply the problem of Bayesian model selection applied to probabilistic graphical models. This approach has been used with remarkable success to explore inductive learning and concept acquisition (Tenenbaum et al., 2011). The issue of how to select the correct hidden variables in the first place has been less well explored, at least in cognitive science (though see Collins and Koechlin, 2012; Gershman and Niv, 2012), but one solution to this problem is provided by Bayesian non-parametric models that entertain, in principle, an infinite model space (Rasmussen and Ghahramani, 2002; Gershman and Blei, 2012).

A clear prediction of structure learning models is that previously acquired structures may be utilized on novel tasks, as manifested by “learning to learn,” where new tasks with the same structure as previously experienced ones are learnt faster. This pattern of behavior has been repeatedly demonstrated in animal experiments (Harlow, 1949; Schrier, 1984; Langbein and Siebert, 2007), as well as those involving human children and adults (Duncan, 1960; Hultsch, 1974; Brown and Kane, 1988; Halford et al., 1998; Acuña and Schrater, 2010), as usefully reviewed in Braun et al. (2010). The same phenomenon has also been rediscovered recently by memory researchers, and described in terms of cognitive schema (Tse et al., 2007; van Kesteren et al., 2012). This means that, given the constraints of their phenotype, adult organisms are likely to have already acquired a large number of possible structures (Kemp and Tenenbaum, 2008; Tenenbaum et al., 2011), which they can use to model the world, and model comparison can thus proceed considering only this reduced model space.

### Signatures of model comparison

An interesting practical question is how we distinguish between separate models, and different parts of a single more complicated model. This is particularly pertinent, because as we have discussed elsewhere (see Supplementary Material, A4 “Free Energy and Model Averaging”), performing variational inference on model probabilities effectively involves embedding them within a larger hierarchical model. On one level, this question is a philosophical one, but in the context of specific cognitive or neuronal hypotheses we take it that what is useful to consider as separate models will generally be fairly clear in terms of functional anatomy [for example, the anatomical dissociation between the neuronal mechanisms underlying goal-directed and habitual behavior discussed earlier (Yin and Knowlton, 2006)]. More concretely, we can point to the fact that complexity plays a key role in adjudicating among different models, but not when weighting different kinds of information within a model (Deneve and Pouget, 2004), and suggest that if behavior shows clear evidence of a bias toward using simple models (as in habit-formation), then this is evidence that model evidence is being used to optimize behavior.

### Active sampling and model comparison

Although—for the sake of simplicity—we have only considered static models in our theoretical discussion, the principles outlined can be easily extended to incorporate extended timeframes and dynamics by minimizing the path-integral of the variational free energy (or the action) over time (Feynman, 1964; Friston, 2008; Friston et al., 2008). Given a particular model, this leads naturally to active sampling of the world in such a way as to minimize uncertainty about its parameters (hypothesis testing) (Friston et al., 2012a). In the context of uncertainty over models, a similar process should occur; with agents actively sampling sensory data in order to disambiguate which model of the world (hypothesis) is best [a beautiful example of this is Eddington's test of general relativity using gravitational lensing (Dyson et al., 1920)]. This notion is supported by recent work showing that in a sequential decision-making context, human subjects trade off reward minimization against gaining information about the underlying structure of the task (Acuña and Schrater, 2010).

### Model comparison and psychopathology

A number of psychiatric disorders are associated with symptoms such as delusions and hallucinations which seem likely to reflect dysfunctional models of their environment (Fletcher and Frith, 2008; Adams et al., 2013; Brown et al., 2013). In some cases this might be the product of pathological learning of the parameters of particular models, but it is also conceivable that impairments in the ability to adequately compare models (to make or utilize inferences about model probabilities) might underlie some deficits. This is also a promising area for future study.

## Summary

In this paper we suggest, based on both theoretical grounds and consideration of behavioral and neuroscientific evidence, that the brain entertains multiple models of its environment, which it adjudicates among using the principles of approximate Bayesian inference. We discussed these principles, which can be implemented in a neurobiologically plausible way using predictive coding (Friston, 2005). Finally, we argue that a number of disparate behavioral and neuroscientific observations are well explained by invoking Bayesian model averaging, focusing particularly on habitual vs. goal-directed control, and why simple models often prevail over more sophisticated ones. We anticipate that this perspective may be useful for hypothesis generation and data interpretation across a number of fields treating both normal function and psychiatric disease.

### Conflict of interest statement

The authors declare that the research was conducted in the absence of any commercial or financial relationships that could be construed as a potential conflict of interest.
