# Supplementary figures and images for: Model averaging, optimal inference, and habit formation
Source: Front Hum Neurosci. 2014 Jun 26;8:457. doi: 10.3389/fnhum.2014.00457 (PMC4071291; doi:10.3389/fnhum.2014.00457)

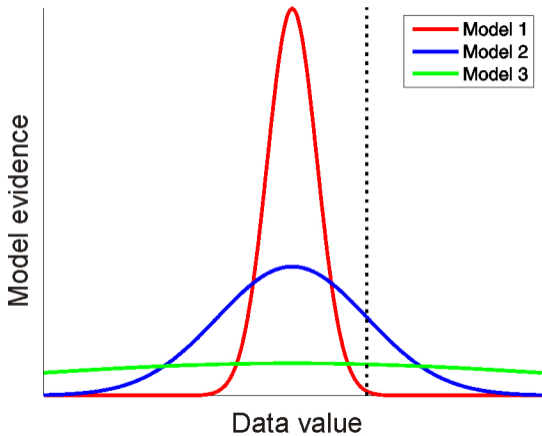

Supplement: Supplementary file 1 [file Presentation1.PDF]
