# Supplementary material for: Model averaging, optimal inference, and habit formation
Source: Front Hum Neurosci. 2014 Jun 26;8:457. doi: 10.3389/fnhum.2014.00457 (PMC4071291; doi:10.3389/fnhum.2014.00457)
Supplement: Supplementary file 2 [file DataSheet1.DOCX]

**Appendix**

**A1 Estimating the evidence for a model**

Consider a situation in which we have a set of observations $y$ and a model $m$ with parameters $\vartheta$ that we are trying to estimate. Here, the model evidence $p(y|m)$ (also sometimes known as the marginal or integrated likelihood) is given by:

$p\left( y | m \right)= \int p(y,\vartheta|m)d\vartheta= \int p(y|\vartheta,m)p\left( \vartheta| m \right)d\vartheta$ A1

This implicitly reflects both the accuracy of the predictions made by the model and the complexity of the model, where the log evidence $L$ can be thought of as having the general form (Box 1 in (Friston, 2010)):

$$L=\log p\left( y | m \right)=\log p\left( y | m \right)\int p\left( \vartheta| y,m \right)d\vartheta=\int p\left( \vartheta| y,m \right)\log p\left( y | m \right)d\vartheta=\int p\left( \vartheta| y,m \right)\log\frac{p\left( y,\vartheta| m \right)}{p\left( \vartheta| y,m \right)}d\vartheta=\int p\left( \vartheta| y,m \right)\log\frac{p\left( y | \vartheta,m \right)p\left( \vartheta| m \right)}{p\left( \vartheta| y,m \right)}d\vartheta=\int p\left( \vartheta| y,m \right)\log p\left( y | \vartheta,m \right)d\vartheta-\int p\left( \vartheta| y,m \right)log\frac{p\left( \vartheta| y,m \right)}{p\left( \vartheta| m \right)}d\vartheta$$

$=Accuracy-\mathrm{Complexity}$ A2

Here, we have used standard results from calculus and Bayes rule to rearrange the expression for log model evidence into the expected likelihood (accuracy) and the Kullback-Leibler divergence between the posterior and prior densities over model parameters (complexity): see (MacKay, 1992). The first term represents how well the model fits the data – given posterior beliefs about the parameters – so this term reflects the accuracy of the model fit to the data. The second complexity term is the Kullback-Leibler divergence between the posterior and prior beliefs. This reflects the difference in the distribution over model parameters before and after observing a set of data. It is this difference that induces a complexity cost and computational load.

To see how this constitutes a penalising complexity term, consider the example of one parameter models with Gaussian priors of different variance (Figure A1). If the variance of the prior is too low, then $p\left( \theta| m \right)$ becomes small rapidly away from the expectation of the distribution. If the variance is high, by contrast, $p\left( \theta| m \right)$ is low because the probability mass is spread too thinly across parameter space. These cases represent models that are too simple and too complex respectively, such that an intermediate model will give the largest value for $p\left( \theta| m \right)$. Complexity can be understood intuitively here as the flexibility of the model, or equivalently, the diversity of data sets it can generate (MacKay, 1992). (In this example, if simulated data were generated from each model, the one with the largest prior variance would tend to create a more varied data set) In a similar vein, it can be easily seen that adding extra parameters will, in general, reduce the probability mass at any given point in parameter space, and thus increase model complexity. Since complexity is always positive (because it is a divergence or distance like measure), it reduces the value of the model evidence, and thus represents a penalty.

**A2 Bayesian model averaging**

The model evidence scores the quality of a model and plays a central role in Bayesian model comparison. Consider the situation where instead of a single model $m$ we have a set of models $\{m_{i}\}$ where $i=1,\ldots,I$ that we wish to compare. Here a posterior distribution $p(m_{i}|y)$ over the set of models is given by:

$p\left( m_{i} | y \right)= \frac{p\left( y | m_{i} \right)p\left( m_{i} \right)}{p\left( y \right)}$ A3

where $p(y)$ is a normalisation constant given by:

$p\left( y \right)=\sum_{i=1}^{I} p\left( y | m_{i} \right)p(m_{i})$ A4

A simple way to make use of this posterior distribution is to perform Bayesian model selection, where the model with the highest posterior probability is selected and used for further inference (see for example (Stephan et al., 2009)). However, the optimal way to make use of this information is to perform Bayesian model averaging which uses a weighted average of the predictions of individual models, with the weight determined by posterior model probabilities (Hoeting et al., 1999; Attias, 2000). Here, if we wish to make a prediction about a new observation $z$ based on our original data and models, then this is given by:

$p\left( z | y \right)= \sum_{i=1}^{I} p\left( z | m_{i},y \right)p(m_{i}|y)$ A5

Similarly, if we wish to predict the value of a parameter $\vartheta$ then this is given by:

$p\left( \vartheta| y \right)= \sum_{i=1}^{I} p\left( \vartheta| m_{i},y \right)p(m_{i}|y)$ A6

This means that the overall posterior distribution is a weighted average of the posterior distributions specified by individual models multiplied by the posterior model probability $p(m_{i}|y)$.

In fact, Bayesian model averaging and selection can be regarded as continuous with one another. To see this, we define:

$K_{i}=\log p\left( y | m_{i} \right)+\log p\left( m_{i} \right)$ A7

and rewrite A3 as a softmax function:

$p\left( m_{i} | y \right)= \frac{e^{{\beta K}_{i}}}{\sum_{j=1}^{I} e^{{\beta K}_{j}}}$ A8

with an inverse temperature parameter $\beta$ that controls the sensitivity of model averaging to which model is best. When $\beta=0$, all models will be treated equally, when $\beta=1$ classic (and optimal) Bayesian model averaging will be performed, and as $\beta\to\infty$ the agent will perform pure model selection.

**A3 Free energy & model evidence**

Although calculating the model evidence is, in general, intractable, the problem can be made easier by the use of an approximating distribution $q(\vartheta|m)$ over model parameters. This distribution can be adjusted to provide the best approximation to the posterior distribution $p\left( \vartheta| y,m \right)$ and – at the same time – a (lower bound) approximation to the log evidence. This adjustment can be expressed as minimising variational free energy, which can be expressed as

$F=- \int q\left( \vartheta|m \right)\ln\frac{p\left( y,\vartheta\right)}{q\left( \vartheta|m \right)}d\vartheta=-ln p(y)+KL(q(\vartheta|m)||p(\vartheta|y))$ A9

where $KL(q(\vartheta|m)||p(\vartheta|y))=\int q\left( \vartheta|m \right)\ln\frac{q\left( \vartheta|m \right)}{p\left( \vartheta| y \right)}d\vartheta$

is the Kullback-Leibler divergence between the approximating distribution $q(\vartheta)$ and the true posterior distribution $p(\vartheta|y,m)$. Since $KL\geq0$ due to the Gibbs inequality, the variational free energy provides a lower bound on (negative) model evidence, the two becoming equal only when the approximating distribution is identical to the true posterior. Variational methods seek to minimise $F$ and in so doing to minimise the KL-divergence (Beal, 2003). This renders the approximating distribution close to the true posterior, essentially killing two birds with one stone: first, by providing a tractable and generic way of implementing approximate Bayesian inference – through the approximate posterior. Second, when minimised, the free energy will approximate the negative log model evidence – enabling Bayesian model selection or averaging.

To ensure that the optimisation of $F$ is tractable, it is usually assumed that $q(\vartheta|m)$ factorises over a partition of parameters (this is called the ‘mean-field assumption’). Generally, one assumes a separation of temporal scales to factorise the distribution $q(\vartheta)$ into a distribution over hidden (time sensitive) variables or causes $q(u)$, (time invariant) parameters linking hidden variables to each other and observed outcomes $q(\theta)$ and hyperparameters governing the precision of random effects $q(\lambda)$, such that (Friston, 2008):

$q\left( \vartheta|m \right)=q\left( u \right)q\left( \theta\right)q(\lambda)$ A10

The variational free energy can now – given appropriate priors and likelihood distributions (Beal and Ghahramani, 2003) – be optimised using Variational Bayes (VB) (Beal, 2003; Attias, 2000) in a series of discrete Bayesian updates or Variational Laplace (Friston et al., 2007) using gradient ascent. Both schemes converge on the same solution; however, the latter is neurobiologically more plausible because it can be formulated as a dynamic process in continuous time.

**A4 Free energy and model averaging**

As well as using the variational free energy to estimate the evidence for each model, we can use it to perform Bayesian model averaging (Attias, 2000; Stephan et al., 2009). Here, we want to approximate the posterior distribution $q(m)\approx p(m|y)$ over models in our model space $m\in M$ in terms of the free energy of individual models $F_{i}\approx-\log p(y|m_{i})$ and any prior beliefs $p(m_{i})$ about their relative plausibility:

$q\left( m_{i} \right)\propto e^{{-F}_{i}}p\left( m_{i} \right)$ A11

Thus by minimising the variational free energy over the whole set of models, an agent can learn not just what the posterior distributions of parameters and hyperparameters for a particular model should be, but also how those beliefs should be weighted relative to one another. If we make the assumption that all admissible models in the model space are equally plausible *a priori*, then we can ignore the prior over models and the Bayesian model averaging in Equations A5 and A6 become:

$p\left( z | y \right)\propto\sum_{i=1}^{I} q\left( z | m_{i} \right)e^{{-\beta F}_{i}}$

$p\left( \vartheta| y \right)\propto\sum_{i=1}^{I} q\left( \vartheta| m_{i} \right)e^{{-\beta F}_{i}}$ A12

In other words, we can use the free energy to adjudicate among the predictions or contributions of different models of the same data. This highlights the central role of the free energy approximation to log evidence. So how might the brain evaluate variational free energy?

**A5 Hierarchical models and predictive coding**

To see how the brain might estimate the variational free energy of different models, we now briefly discuss the implementation of variational Bayes using neurobiologically plausible models of predictive coding – a subject that has been treated at length elsewhere (Friston, 2005, 2008; Bastos et al., 2012; Mumford, 1992; Rao and Ballard, 1999). For the sake of simplicity, we only consider the case of static models, but this formulation can be easily extended to include dynamics through the use of generalised coordinates of motion (Friston et al., 2008; Friston, 2008). We start by considering a hierarchical model, consistent both with the environment the brain has to model and known features of neuroanatomy (Felleman and Van Essen, 1991; Friston, 2005; Markov and Kennedy, 2013). This model has the following structure

$y=g\left( u^{\left( 1 \right)},\theta^{(1)} \right)+z^{\left( 1 \right)}$ A13

$$u^{\left( 1 \right)}={g(u}^{\left( 2 \right)},\theta^{(2)})+z^{\left( 2 \right)}$$

$$\vdots$$

Here, observed data $y$ are determined by a nonlinear function $g\left( u^{\left( 1 \right)},\theta^{(1)} \right)$ of hidden variables at the first level of the model $u^{\left( 1 \right)}$ and parameters $\theta^{(1)}$ plus a stochastic term $z^{\left( 1 \right)}$. Hidden variables $u^{\left( 1 \right)}$ are determined in a similar way by a nonlinear function ${g(u}^{\left( 2 \right)},\theta^{(2)})$of variables and parameters at the second level of the model, and so on up the hierarchy. Thus variables which are inferred on at one level of the model determine the prior probabilities of variables at the level below (they are thus commonly known as ‘empirical priors’) (Efron and Morris, 1973). If we further assume that the stochastic terms are Gaussian with a covariance given by the hyperparameters $\lambda^{(i)}$ (Friston, 2005), then the variational free energy is given by

$$F=\frac{1}{2}\sum_{i} ({\varepsilon^{\left( i \right)T}\Pi}^{\left( i \right)}\varepsilon^{\left( i \right)}+ln\Pi^{\left( i \right)})$$

$\varepsilon^{(i)}=u^{(i)}-g\left( u^{(i+1)},\theta^{(i+1)} \right)$ A14

where ${\Pi^{(i)}=(\lambda^{\left( i \right)})}^{-1}$ encodes the precision (or inverse covariance) of the random fluctuations $z^{\left( i \right)}$. Thus the free energy is a function of the precision-weighted sum of squared prediction errors and minimising free energy corresponds to minimising (precision weighted) prediction error.

In neuronal models $\varepsilon_{i}$ are termed error units, which send prediction errors to the level above. These units receive backwards and lateral connections from the representational units $u^{\left( i \right)}$encoding the hidden variables (Friston, 2005). Based on the distinctive connectivity profile of different cortical layers, it is thought that prediction errors are encoded by the superficial pyramidal cells and predictions (representations) by pyramidal cells in deep layers (Friston, 2005; Mumford, 1992) – though more detailed models have recently been put forward that include hidden states with temporal dynamics (Bastos et al., 2012). In this formulation, perceptual inference involves minimising precision-weighted prediction errors by dynamically updating the activity of representation units, whilst learning of the parameters and precisions occurs at a slower time scale and may be mediated by changes in synaptic weights and the action of classical neuromodulators (Moran et al., 2013; Friston et al., 2012; Feldman and Friston, 2010).

If the brain entertains different models of the same features of its environment, then these must make predictions about the same data which might be either low level sensory representations or higher level ones (for example at the level of whole objects). For the purposes of this discussion, we will assume that $y$ corresponds to representations at the level in the cortical hierarchy at which descending predictions from different models converge. Formally, we can now entertain the possibility that the brain computes Bayesian model averages of its predictions using the posterior beliefs about different models from Equation A12. Crucially, these posterior beliefs are functions of free energy which – as shown in Equation A14 – is just a function of accumulated prediction errors and their precision.

**FIGURE A1: Graphical illustration of the model evidence (probability of data under a given model). Here, we consider a single datum (dotted line), and the ensuing model evidence under three Gaussian models with the same expectation, but with different variances. Model one (red) has low variance (low complexity), but does not fit the data well and thus has a low accuracy. Model three (green) has high variance (high complexity), which allows it to fit the data, but spreads its probability mass over a large range of values meaning it also has a low model evidence. Thus model two, which balances accuracy and complexity appropriately for this datum, has the strongest evidence (adapted from** (MacKay, 1992)**).**
